# Supplementary material for: Everything, everywhere, all at once - Surveillance and molecular epidemiology reveal Melissococcus plutonius is endemic among Michigan, US beekeeping operations
Source: PLoS One. 2025 Sep 12;20(9):e0331903. doi: 10.1371/journal.pone.0331903 (PMC12431213; doi:10.1371/journal.pone.0331903)
Supplement: S1 Table — Yards were numbered in the order they were sampled and differed for each operation between years. (DOCX) [file pone.0331903.s001.docx]

|  |  | **Migratory Beekeeper ID** (*n=599*) | | | | | | | **Stationary Beekeeper ID** (*n=256)* | | | | | | | | |
| --- | --- | --- | --- | --- | --- | --- | --- | --- | --- | --- | --- | --- | --- | --- | --- | --- | --- |
|  |  | **A** | **C** | **D** | **E** | **F** |  |  | **B** | **G** | **H** | **I** | **J** |  |  |  |  |
| **2021** : *n=358* | Yard 1 | 7/10 | 6/10 | 1/10 | 0/10 | 2/10 |  |  | 1/7 | 3/9 | 1/6 | 1/7 | 0/5 |  |  |  |  |
|  | Yard 2 | 6/10 | 7/10 | 2/10 | 4/10 | 3/10 |  |  | 2/7 | 0/4 | 2/6 |  | 0/5 |  |  |  |  |
|  | Yard 3 | 5/10 | 1/10 | 0/10 | 1/10 | 2/10 |  |  | 2/9 | 3/3 |  |  | 0/5 |  |  |  |  |
|  | Yard 4 | 8/10 | 5/10 | 0/10 | 2/9 | 5/10 |  |  | 0/8 |  |  |  | 2/5 |  |  |  |  |
|  | Yard 5 | 3/10 | 7/10 | 0/10 | 0/10 | 4/10 |  |  | 3/9 |  |  |  | 1/4 |  |  |  |  |
|  | Yard 6 | 8/10 |  |  |  |  |  |  |  |  |  |  |  |  |  |  |  |
| **Total** | | **37/60** | **26/50** | **3/50** | **7/49** | **16/50** |  |  | 8/40 | **6/16** | **3/12** | **1/7** | **3/24** |  |  |  |  |
|  |  | **A** | **C** | **D** | **E** | **F** | **K** | **X** | **B** |  | **H** | **I** | **J** | **L** | **M** | **N** | **O** |
| **2022 :** *n=497* | Yard 1 | 4/10 | 13/23 | 2/10 | 7/10 | 1/10 | 1/19 | 1/8 | 3/8 |  | 0/5 | 8/17 | 0/5 | 0/4 | 2/6 | 2/9 | 7/16 |
|  | Yard 2 | 3/10 | 17/18 | 3/10 | 8/10 | 4/10 | 5/10 |  |  |  | 0/5 |  | 0/5 | 2/4 | 2/5 | 1/4 |  |
|  | Yard 3 | 3/10 | 19/26 | 6/10 | 2/9 | 5/10 | 1/10 |  |  |  | 0/5 |  | 0/5 | 1/4 | 0/5 | 1/5 |  |
|  | Yard 4 | 5/9 | 22/28 | 8/10 | 0/10 | 8/10 |  |  |  |  | 0/5 |  | 0/5 | 0/4 |  | 2/5 |  |
|  | Yard 5 | 4/10 |  | 6/10 | 0/10 | 4/10 |  |  |  |  | 3/5 |  | 0/5 |  |  | 3/11 |  |
| **Total** | | **19/49** | **71/95** | **25/50** | **17/49** | **22/50** | **7/39** | **1/8** | **3/8** |  | **3/25** | **9/17** | **0/25** | **3/16** | **4/16** | **9/34** | **7/16** |
| **Grand Total** | | **56/109** | **97/145** | **28/100** | **24/98** | **38/100** | **7/39** | **1/8** | **11/48** | **6/16** | **3/37** | **9/24** | **3/49** | **3/16** | **4/16** | **9/34** | **7/16** |

**S1 Table.**
