# Supplementary material for: Everything, everywhere, all at once - Surveillance and molecular epidemiology reveal Melissococcus plutonius is endemic among Michigan, US beekeeping operations
Source: PLoS One. 2025 Sep 12;20(9):e0331903. doi: 10.1371/journal.pone.0331903 (PMC12431213; doi:10.1371/journal.pone.0331903)
Supplement: S2 Table — (DOCX) [file pone.0331903.s002.docx]

**S2 Table.**

| **Operation Label** | **Home State** | **Yards sampled** | **Colonies sampled** | **PCR Positive** | **Clinical Disease** |
| --- | --- | --- | --- | --- | --- |
| A | Michigan | 3 | 15 | 1 | 1 |
| C | Michigan | 1 | 5 | 1 | 1 |
| D | Michigan | 1 | 5 | 0 | 0 |
| E | Michigan | 1 | 5 | 0 | 0 |
| F | Michigan | 4 | 19 | 3 | 1 |
| AB | North Dakota | 1 | 5 | 0 | 0 |
| AC | North Dakota | 1 | 5 | 1 | 0 |
| AD | Texas | 1 | 5 | 0 | 0 |
| AE | North Dakota | 1 | 5 | 0 | 0 |
| AF | Texas | 1 | 5 | 0 | 0 |
| AG | Oregon | 1 | 5 | 4 | 5 |
| AH | Michigan | 1 | 5 | 0 | 0 |
| AI | Ohio | 1 | 5 | 0 | 0 |
| AJ | Michigan | 1 | 5 | 0 | 0 |
| AK | Idaho | 1 | 5 | 0 | 0 |
| AL | Oregon | 1 | 5 | 0 | 0 |
| AM | Idaho | 1 | 5 | 0 | 0 |
| AN | Oregon | 1 | 5 | 3 | 2 |
| AO | Idaho | 1 | 5 | 0 | 0 |
| AP | California | 2 | 10 | 0 | 0 |
| AQ | Georgia | 2 | 10 | 0 | 0 |
| AR | No data | 1 | 5 | 1 | 0 |
| **Total** |  |  | **144** |  |  |
