# Supplementary material for: Everything, everywhere, all at once - Surveillance and molecular epidemiology reveal Melissococcus plutonius is endemic among Michigan, US beekeeping operations
Source: PLoS One. 2025 Sep 12;20(9):e0331903. doi: 10.1371/journal.pone.0331903 (PMC12431213; doi:10.1371/journal.pone.0331903)
Supplement: S3 Table — (DOCX) [file pone.0331903.s003.docx]

**S3 Table.**

| Gene | Duplex PCR (Arai et al. 2014)[1] | | Amplicon size (bp) |
| --- | --- | --- | --- |
| Na^+^/H^+^ antiporter | Typical-F | 5’ – TGG TAG CTT AGG CGG AAA AC - 3’ | 424 |
|  | Typical-R | 5’ – ﻿TGG AGC GAT TAG AGT CGT TAG A - 3’ |  |
| Fur family transcriptional regulator | Atypical-F | 5’ – ﻿GAG AAC GAT TCG GTA CAA GC - 3’ | 187 |
|  | Atypical-R | 5’ – ﻿CCT TTT CTT CAC ATT CTG GAC AT - 3’ |  |
|  | | | |
|  | Extracted DNA confirmation (Govan et al. 1998) [2] | |  |
| 16S RNA | Govan-F | 5’ – GAA GAG GAG TTA AAA GGC GC - 3’ | 810 |
|  | Govan-R | 5’ – TTA TCT CTA AGG CGT TCA AAG G - 3’ |  |
