# Supplementary material for: Everything, everywhere, all at once - Surveillance and molecular epidemiology reveal Melissococcus plutonius is endemic among Michigan, US beekeeping operations
Source: PLoS One. 2025 Sep 12;20(9):e0331903. doi: 10.1371/journal.pone.0331903 (PMC12431213; doi:10.1371/journal.pone.0331903)
Supplement: S4 Table — Home state represents the primary state where the beekeeping operation resides. (DOCX) [file pone.0331903.s004.docx]

**S4 Table.**

|  | **Reference (qPCR)** | |  |
| --- | --- | --- | --- |
|  | **Positive** | **Negative** | **Total** |
| **Duplex PCR Positive** | 67 | 2 | **69** |
| **Duplex PCR Negative** | 2 | 2 | **4** |
| **Total** | **69** | **4** | **73** |
