# Supplementary figures and images for: Everything, everywhere, all at once - Surveillance and molecular epidemiology reveal Melissococcus plutonius is endemic among Michigan, US beekeeping operations
Source: PLoS One. 2025 Sep 12;20(9):e0331903. doi: 10.1371/journal.pone.0331903 (PMC12431213; doi:10.1371/journal.pone.0331903)

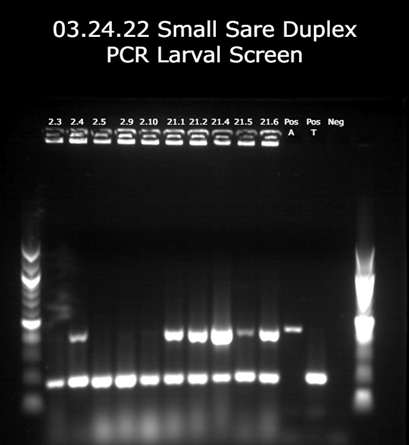

Supplement: S1 Fig — Supplement.zip – Bioinformatics metadata for sequences used in this study. (TIF) [file pone.0331903.s005.tif]
